# Protecting the developing brain by 17βestradiol

**DOI:** 10.18632/oncotarget.14979

**Published:** 2017-02-01

**Authors:** Julien Pansiot, Jérome Mairesse, Olivier Baud

**Affiliations:** Réanimation et Pédiatrie Néonatales & INSERM U1141, Hôpital Robert Debré, Paris, France

**Keywords:** neuroprotection, myelination, estrogens, developing brain, repair, Neuroscience

Among estrogen hormones, 17β-estradiol (17βE2) is the most potent natural form in circulation in mammals. It is known for its role in fetal development, fertility, sexual dimorphism, bone structure, and also for its role in breast or other hormone-dependent cancers. It has also remarkable effects on several systems including the cardiovascular, immune, and nervous systems [1].

17βE2 is a lipophilic steroid hormone that can diffuse across the blood-brain barrier. It has been extensively studied as a neuroprotective molecule in several preclinical models of brain damage mimicking stroke, hypoxia, excitotoxicity, inflammation, traumatic injury. The use of both *in vivo* and *in vitro* models has demonstrated that 17βE2 exerts many actions on all the different cell types populating the developing brain, including notably immature oligodendrocytes, microglia, astrocytes and neurons. In a preclinical model of excitotoxic brain injury developed in rodents, we demonstrate that 17βE2 exposure significantly reduces lesion size, astrocyte-mediated neuronal cell death and white matter damage mediated by neuroinflammation [2]. These data provide evidence for an *in vivo* neuroprotective effect of 17βE2 in a rat model mimicking periventricular leukomalacia, a common condition observed in very preterm infants.

In oligodendroglial lineage, 17βE2 promotes the proliferation of immature oligodendrocytes, their differentiation into myelinating oligodendrocytes and strongly reduces apoptotic cell death in response to noxious insult. 17βE2 reduces neuro-inflammation, known to disrupt oligodendroglial maturation, by downregulating gene transcription of inflammatory mediators and cytokines including inducible nitric oxyde synthase, interleukin-1β, interleukin-6, TNF-α and reactive oxygen species in microglial cells. More precisely, this hormone can switch microglial cells from a pro-inflammatory phenotype M1 to an anti-inflammatory phenotype M2. Hormonal effect on astroglial cells is also significant [3]. 17βE2 is involved in astrogliosis by decreasing astrocytic proliferation, glial fibrillary acidic protein and vimentine expression in injured brain. Furthermore, many molecules involved in glial response to brain injury are regulated by 17βE2: in the astrocytes, several studies showed a decrease of pro-inflammatory cytokines, cyclo-oxygenase 2, nitric oxide, NF-κB, and aquaporin 4, while some growth factors (TGFα, TGFβ1, TGFβ2, FGF2, GDNF, BDNF, IGF-1) are increased as well as the glutamate transporters (GLT-1, GLAST). Altogether, these changes lead to a reduction in brain inflammation, excitotoxicity and neuronal cell death.

17βE2 could induce either direct or indirect actions through cellular crosstalk in brain structures. Some *in vitro* studies have demonstrated that 17βE2 has a direct protective action on neuronal cell survival. But it has been also proposed that this property could be largely mediated by astrocytes releasing many growth factors and the decreasing extracellular glutamate levels. Estrogen receptors named ERα, Erβ play also a key role to mediate these properties. ERs are widely expressed in the brain where they are localized in the neurons and the glial cells. 17βE2 effects can be mediated by ERs via genomic and non-genomic pathways through ERs. For genomic effects, 17βE2 passively diffuses through the plasma membrane, binds ERs in the cytoplasm, translocates to the nucleus and binds specific DNA sequences called estrogen response elements to regulate genes encoding pro-survival factors and pro-apoptotic proteins. For non-genomic effects, 17βE2 acts more rapidly by binding ERs located on the plasma membrane and regulates kinase signaling pathways including MAPK/ERK and PI3K/Akt that are involved in the regulation of transcription factors, nuclease kinases, survival, adhesion, metabolism and cell proliferation.

17βE2 beneficial effect could be mediated by direct effect on central nervous system or peripheral effects. Studies showed that Erα is mostly involved in neuroprotective central effect of 17βE2. In particular, signalling through ERα in astrocytes, but not through ERα in neurons, appears essential for the beneficial effects of ERα ligand in experimental autoimmune encephalomyelitis [4]. These findings could have implications for understanding the pathophysiology of sex hormone effects in diverse CNS disorders including developmental brain damages. Nevertheless, it has been shown that the mechanisms involved in the protective effect of 17βE2 are not always mediated by ERs. It could be observed through membrane G protein-coupled estrogen receptor (GPR30, ER-X) with a faster action after estadiol binding. In addition to the regulation of genes transcription, 17βE2 enables to mediate also the expression of miRNA which are relevant during neuropathological processes of ischemia of other brain injury [5].

Finally, 17βE2 could have peripheral actions because ERs are expressed in different organs. Alternatively to 17βE2, selective estrogen receptor modulators (SERMs, including tamoxifen, raloxifene that are currently used in clinical practice) could be used to confer neuroprotection with a potent effect on inflammation and astrogliosis, regulation of glutamate transporters and release of neurotrophic factors. SERMs may be promising candidate as neuroprotective therapies, but their added-value in clinical practice for this purpose remains to be demonstrated. In particular, long-lasting effects of 17βE2 given during perinatal period should be documented considering putative neuro-endocrine and behavioural effects of this hormone on the developing brain.

## References

[1] Arevalo (2010). Biochim Biophys Acta.

[2] Pansiot (2016). Exp Neurol.

[3] Johann (2013). J Steroid Biochem Mol Biol.

[4] Spence (2011). Proc Natl Acad Sci U S A.

[5] Herzog (2016). J Steroid Biochem Mol Biol.

